# Experimental Interrogations: *Tatbestandsdiagnostik*, Objectivity, and the Impact of Experimental Psychology on Early-Twentieth-Century Criminal Justice

**DOI:** 10.1007/s00048-025-00431-7

**Published:** 2025-10-17

**Authors:** Elwin Hofman

**Affiliations:** Utrecht University, Utrecht, The Netherlands

**Keywords:** Psychology, Association tests, Knowledge circulation, Criminal justice, Objectivity, Subjectivity, Psychologie, Wortassoziation, Wissenszirkulation, Strafrecht, Objektivität, Subjektivität

## Abstract

In 1904, Max Wertheimer and Julius Klein published a paper that shook the worlds of criminal justice and psychology. They proposed using psychological experiments, particularly word association tests, to assess whether criminal suspects had committed a particular crime. Over the following months and years, almost every German-language journal on psychology or criminal law, as well as many foreign-language journals, published something on this so-called *Tatbestandsdiagnostik*. Some hailed it as the “criminal investigation of the future.” However, Tatbestandsdiagnostik’s downfall was as swift as its rise to fame. By the advent of World War I, most psychologists and jurists had concluded that the association method was of no use in legal and police practice. This article traces the history of Tatbestandsdiagnostik as a case of how new forms of psychological knowledge circulated, were evaluated, and made an impact. It argues that proponents’ insistence on the method’s objectivity, its ambiguous relationship with psychoanalysis, and the possibility of demonstrating it to students and colleagues facilitated both its rapid rise and its demise.

## Abstract (de)

Im Jahr 1904 veröffentlichten Max Wertheimer und Julius Klein eine Abhandlung über die „Tatbestandsdiagnostik“, die die Welt der Strafjustiz und der Psychologie im Sturm eroberte. Die Autoren schlugen vor, psychologische Experimente, insbesondere die Methode der Wortassoziation, bei Tatverdächtigen anzuwenden, um ihre Schuld zu prüfen. In den folgenden Monaten und Jahren veröffentlichten fast alle deutschsprachigen psychologischen und strafrechtlichen Fachzeitschriften und auch viele fremdsprachige Zeitschriften zu diesem Thema. Einige Autoren sahen in der Tatbestandsdiagnostik die „Strafuntersuchung der Zukunft“. Doch so schnell, wie die Tatbestandsdiagnostik die Welt eroberte, verschwand sie auch wieder. Zu Beginn des Ersten Weltkrieges kamen die meisten Psychologen und Juristen zu dem Schluss, dass die Assoziationsmethode in der juristischen und polizeilichen Praxis unbrauchbar sei. Dieser Artikel zeichnet die Geschichte der Tatbestandsdiagnostik als ein Beispiel dafür nach, wie neue Formen psychologischen Wissens zirkulierten, bewertet wurden und Auswirkungen hatten. Es wird argumentiert, dass das Beharren der Befürworter der Tatbestandsdiagnostik auf der Objektivität der Methode, ihre unklare Beziehung zur Psychoanalyse und die Möglichkeit, die Methode Studenten und Kollegen zu demonstrieren, sowohl ihren raschen Aufstieg als auch ihren Niedergang begünstigten.

## Introduction: A Victory for Justice and Psychology

**Fig. 1:**
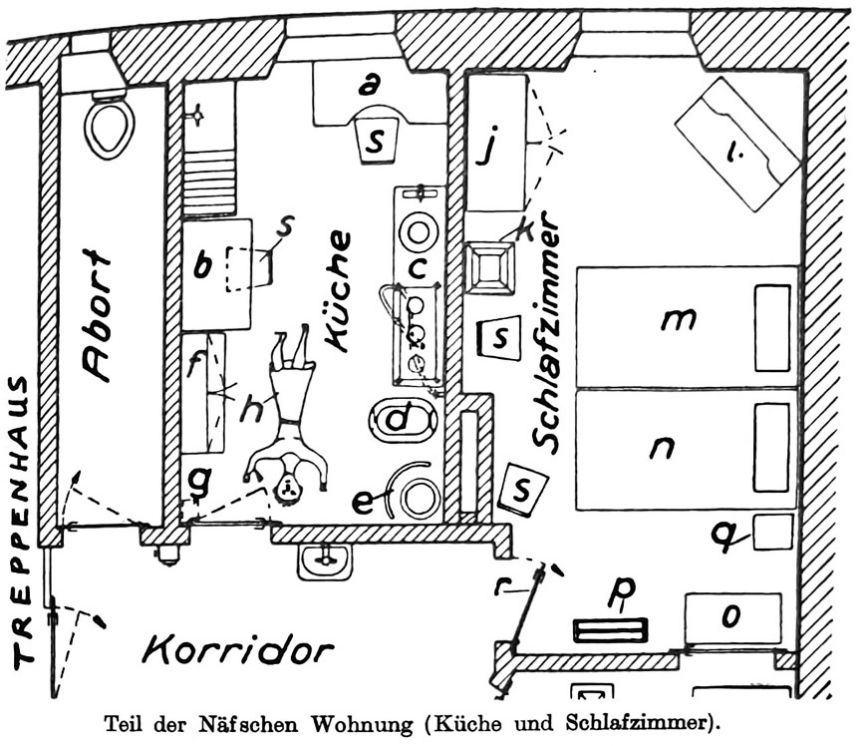
Sketch of the crime scene in the Näf case, published in *Spiegel* (1937: 101)

At around half past seven on the morning of February 22, 1934, desperate cries were heard in the stairwell of a rented property in Zurich.^1^ The cries came from Hans Näf, a dental technician. When neighbors arrived at the apartment, they found his wife Luise’s lifeless body on the kitchen floor (Fig. 1). A strange smell pervaded the room. It quickly became clear that Luise Näf had died from gas. But was it an accident, suicide, or murder? Suspicions arose against Hans Näf, who was set to collect a significant sum of money from Luise’s life insurance. Näf denied everything, but evidence was mounting against him. He was charged with murder. Although most of the evidence against Näf was circumstantial, the difficulty lay in his person: he was antisocial and had previous convictions for theft and possession of narcotics. Hoping to find some evidence of his innocence in his psyche as well, the defense asked the court to request an expert to weigh in: the psychoanalyst Carl Gustav Jung.

Busy as he was, Jung was delighted that his expertise had been asked for by the Zurich court (Jung & Neumann 2015: 81). Jung took this request as a chance to apply a peculiar psychological technique called Tatbestandsdiagnostik. Jung’s version of this method consisted of providing the suspect with a series of keywords, to which he had to respond as quickly as possible with the first word he thought of. Many of these words were irrelevant and only served as benchmarks. However, some of the keywords related to the crime or its circumstances. Committing a crime, Jung argued, left unconscious traces in the mind; it created psychic complexes. Hence, if criminals were confronted with words relating to a crime they had committed, they would either betray themselves with their association (for example, “wallet—blue” in a case where a blue wallet had been stolen) or because their reaction was delayed or nonsensical.

Jung subjected Näf to a series of 407 keywords, based on the indictment he had received. Of these 407 words, 271 were neutral, 96 related to the evidence, and 40 related to the defendant’s emotional life. After a session of more than three hours, Jung concluded that Näf was guilty. Näf’s reactions were generally slow, even slower than the average for uneducated people. Two thirds of the critical keywords led to “disturbances,” which never occurred with the neutral keywords. All these findings, Jung argued, pointed towards clear feelings of guilt (Jung 1937; Jung & Neumann 2015: 81–82n254). Jung testified before the jury and, as he later proudly told the British newspaper *The Daily Mail*, Näf “was, of course, convicted and sentenced” (Word Clues 1935). A victory, it seemed, for justice and psychology. But, as we will see, the case was not yet closed.

Jung’s use of Tatbestandsdiagnostik in the Näf case was one of the most prolific uses of the method. Yet by 1934, Tatbestandsdiagnostik was by no means new. Rather to the contrary: the method was already three decades old and two decades past its prime. Its history dates back to 1904, when two enterprising students, Max Wertheimer and Julius Klein, published a paper that shook the worlds of criminal justice and psychology. They proposed applying psychological experiments, such as the word association test, to criminal suspects to determine their involvement in a particular crime. They christened this new method of criminal investigation Tatbestandsdiagnostik (Wertheimer & Klein 1904).

Wertheimer and Klein’s paper was mostly programmatic; it contained few empirical results. But shortly after its publication in Hans Gross’s well-read journal on criminalistics, both psychologists and jurists started to respond. Some were enthusiastic, others critical. Every noteworthy German-language journal on psychology or criminal law published something on the matter. Some hailed Tatbestandsdiagnostik as the “criminal investigation of the future” (Rittershaus 1912). However, Tatbestandsdiagnostik’s downfall was as swift as its rise to fame. By the advent of World War I, most psychologists and jurists concluded that while the association method was psychologically interesting, it was of no use in legal and police practice. Jung was a lone exception.

In this article, I study the rise and fall of Tatbestandsdiagnostik as a case of how new forms of psychological knowledge circulated, were evaluated, and made an impact—or lacked such an impact. Today, Tatbestandsdiagnostik is all but forgotten. Yet its brief scientific career is revelatory of the histories of psychology and criminal justice in the early twentieth century. Tatbestandsdiagnostik shows how psychology tried to establish itself as a useful scientific discipline. It highlights how enthralled people were with promises of greater objectivity around the turn of the twentieth century. As Tatbestandsdiagnostik was readied for practice, however, enthusiasm about objectivity quickly started to wane. By the time of World War I, magistrates and prosecutors were increasingly convinced that their trade required subjective appreciation, not objective experiments. Experimental psychologists, meanwhile, found that Tatbestandsdiagnostik was not yet objective enough. Still, belief in Tatbestandsdiagnostik lasted the longest, I will show, among those like Jung, who appreciated its subjective, psychoanalytic elements. As such, Tatbestandsdiagnostik became one of the issues that allowed scholars to discuss the values of objectivity and subjectivity in their discipline. The brief career of Tatbestandsdiagnostik highlights how important these discussions were in the early twentieth century.

Insofar as Tatbestandsdiagnostik has been studied, scholars have framed it within the history of German forensic psychology. Tatbestandsdiagnostik, in this reading, was part of psychologists’ efforts to show the applicability of experimental psychology in—and their own necessity to—criminal justice (Wolffram 2018: 77–78; Schmoeckel 2009: 73–75; Lück & Niehaus 2007: 151–61). But I will show that there is more to it than this. With this article, I want to study this method as a means of bringing together three historiographical lines of inquiry that are often studied separately: the history of knowledge circulation, the history of objectivity in psychology, and the history of practical or applied knowledge. I argue that knowledge about Tatbestandsdiagnostik circulated so rapidly and so widely because it seemed to solve two of the key issues psychologists struggled with at the time: studying the mind objectively and demonstrating the practical use of their discipline. However, when Tatbestandsdiagnostik could not surpass the tension between these two goals, it quickly fell into obscurity.

In their history of objectivity in scientific atlases, Lorraine Daston and Peter Galison (2007) observed three “codes of epistemic virtue” relating to how scientists represented reality. The oldest, “truth to nature,” entailed idealization of scientific phenomena, stripping away accidental observations to find what was essential. It was questioned in the nineteenth century, when scientists increasingly aimed to erase subjectivity and simply present findings as they had been registered by a machine, without interpretation or abstraction. Daston and Galison called this ideal “mechanical objectivity.” By the early twentieth century, they argue, this approach became less popular again, in favor of what they call “trained judgment.” The results of the machines had to be interpreted with a scientifically trained eye, signaling the return of intuition and subjectivity to scientific practice.

Daston and Galison’s thesis connects to a longer-standing debate on a tension visible in psychology and many other scientific disciplines around 1900, a tension between positivist desires for objectivity on the one hand and hermeneutic appreciations of subjectivity on the other (Smith 2013: chap. 5; Dror 1999). These tensions, I will show, largely determined the fate of Tatbestandsdiagnostik. Because the method could incorporate both mechanical registration and intuition, discussions about objectivity and subjectivity could crystallize around it. More than in the purely scientific debates Daston and Galison discussed, experience in life—rather than with scientific experiments—became the key alternative to mechanical objectivity. At the same time, these discussions allow me to nuance the all-too-stark focus that has been put on increasing objectivity in analyses of forensic science around 1900.^2^

To support my argument, I mainly rely on scientific publications, reviews, conference reports, bibliographies, and newspaper reports and announcements. I use them not just for the information they contain about Tatbestandsdiagnostik, but also take them seriously as artefacts of knowledge circulation.^3^ They are remnants of the material, infrastructural, and institutional means by which knowledge circulated. They reveal the trajectories Tatbestandsdiagnostik followed between different places and between different groups of people, including psychologists, psychiatrists, legal scholars, and practicing magistrates. Moreover, to assess the circulation, evaluation, and impact of this new scientific technique, I take into account not only German-language, but also Dutch, French, Italian, and American contributions and interventions. As such, we also get a better understanding of Germany’s—and the German-speaking world’s—predominant role in early-twentieth-century (forensic) psychology.

## 1. Inventing a New Method

Wertheimer and Klein’s Tatbestandsdiagnostik built on recent advances in psychology and psychiatry. Conducting psychological research through experiments, preferably in laboratories, had become the key to scientific knowledge about the human mind in the German-speaking world in the last decades of the nineteenth century. Psychologists established their own laboratories and institutes at universities, publicized their findings at new conferences and in new journals, and sometimes attracted a wider audience with public demonstrations of their experiments (Smith 2013: 79–93). Experimental psychologists developed or learned to use all sorts of technical instruments to observe physiological reactions with scientific precision, including chronometers to measure reaction times, pneumographs to measure breathing, and sphygmographs to measure pulse.

Most famous for this new psychological method was undoubtably Wilhelm Wundt, whose psychological laboratory in Leipzig, established in 1879, attracted and inspired numerous visitors, even from far outside Germany (Danziger 1980). Stimulated by experiments of the British statistician Francis Galton, one of the first psychological experiments Wundt conducted was a word association test. He found that when people were asked to react to a certain word with another word as quickly as possible, their reactions were often similar and dependent on experiences in their early youth. Wundt and his students also measured reaction times and studied various factors affecting the speed of a response (Mülberger 2017: 181–82; Herbold-Wootten 1982: 248–49). These first experiments were followed by a period of disillusion: after many experiments, it seemed that people’s immediate reactions to a word did not reveal all that much about their psyche (L. Bouman 1908: 24). But early-twentieth-century psychiatrists, most notably Carl Gustav Jung, built on these findings and suggested that aberrant reactions or lengthened reaction times could reveal psychic “complexes,” unconscious clusters of emotions, memories, and perceptions. Influenced by Sigmund Freud and psychoanalysis, they found that experimental psychological techniques could hence make unconscious mental activity visible (Jung 1906a; 1906c: 145–60; Freud 1906: 1–2). Objective techniques therefore proved to be useful even to study subjective matters.

Many criminal jurists followed the advances in psychology with interest. Since the early nineteenth century, stimulated by enterprising psychologists, especially German criminalists had taken an interest in psychology as a means to improve the effectivity of both criminal investigation and criminal punishment (Hofman 2022; Greve 2004). This interest seems to have waned somewhat by the mid-nineteenth century, but it returned strongly as the new techniques of experimental psychology emerged in the century’s closing decades. The Italian criminologist Cesare Lombroso, for instance, made use of several psychometric instruments in his work on born criminals from the 1880s onwards (Horn 2003: 125–29; Bunn 2019: 151–52). Around 1900, Franz von Liszt, an influential criminologist and professor of criminal law in Berlin, included psychological insights in his classes for law students and invited psychologists in his seminars to present experiments on the reliability of memory and eyewitnesses (Mülberger 2009b: 64, 69; Schmoeckel 2009: 71–73). Yet the emblem of the interest in psychology among criminalists was Hans Gross, the “father of forensic psychology” in German-speaking Europe. Gross had extensive experience as an investigating judge in Austria before becoming a professor in 1897. The following year, he published *Criminalpsychologie*, an overview of the psychological knowledge that judges needed. The book frequently referenced Wundt and other experimental psychologists (Wolffram 2018: 61–70). By the beginning of the twentieth century, local interdisciplinary societies for forensic psychology had been established in several German and Austrian cities.^4^

It was through Gross’s classes in Prague that Wertheimer and Klein came in touch with the forensic applications of psychology. Wertheimer had been studying law in Prague, where he met Klein, a fellow law student. They started their work on Tatbestandsdiagnostik under the auspices of Gross in 1902. Wertheimer then switched to philosophy to focus more on psychology (a subfield of philosophy at the time). After writing his initial article with Klein, Wertheimer would conduct experimental studies on Tatbestandsdiagnostik in the laboratory of Oswald Külpe in Wurzburg, where he obtained his PhD in 1904 (Newman 1944: 429–30; Michael Wertheimer et al. 1992: 47–48). Wertheimer continued to engage with the debates on Tatbestandsdiagnostik afterwards, while Klein disappeared from the scientific forum.^5^

Most studies in forensic psychology around 1900 focused on the reliability of witnesses, but Wertheimer and Klein redirected their attention to suspects. Unlike earlier studies that had attended to the psychology of suspects, they did not attempt to convince suspects to confess by playing to their emotions. Instead, they looked to the more objective, empirical techniques of experimental psychology (Wertheimer & Klein 1904: 74–76). Wertheimer and Klein mainly highlighted the associational test, which seems to have been a practical choice. They suggested other possibilities for Tatbestandsdiagnostik, including using psychodiagnostic instruments to measure emotional responses to stimulus words. But they only experimented themselves with the association test, which only required a chronometer, a pencil, and a list of words. This made the method useable for students such as themselves, who did not have access to a psychological laboratory. It also goes some way in explaining why this technique became popular so quickly: it was relatively easy to try out. The association test would soon become synonymous with Tatbestandsdiagnostik (Wertheimer & Klein 1904: 76–90).

If association tests could reveal psychic complexes and repressed memories, Wertheimer and Klein asked, would they not be able to reveal the psychic consequences of committing a crime? They carried out some preliminary experiments with associations and reaction times (on one another and on fellow students), and these seemed to answer this question affirmatively. Through perception and experience, Wertheimer and Klein argued, committing a particular act left a certain “complex” in the mind, an association of people, circumstances, events, judgments, and feelings, especially if strong emotions or interests were involved. As a result, people’s immediate associations with relevant words changed. To reveal such complexes, Wertheimer and Klein proposed to confront people with a series of stimulus words, some of them meaningless, others critical, related to the event in question, and to ask them to react immediately. When confronted with critical stimulus words, people guilty of a particular crime would reveal themselves either by their response, or, when avoiding giving an incriminating response, by their increased reaction time (Wertheimer & Klein 1904: 72–73, 110–13). If their initial findings were confirmed on a larger scale, Wertheimer and Klein’s proposal promised to herald a new approach to criminal interrogation.

## 2. Tatbestandsdiagnostik Encounters the World

Wertheimer and Klein’s Tatbestandsdiagnostik appeared at just the right moment to make an impact. The recent successes in witness psychology had made jurists attentive to innovations with psychological experiments. A series of journals with a distinct interest in crime and psychology had recently been established: Hans Gross had started publishing a journal on criminalistics in 1899 (in which Wertheimer and Klein naturally published their article), William Stern a journal on witness psychology in 1903, Gustav Aschaffenburg a journal on criminal psychology in 1904.^6^ Franz von Liszt’s longer-running and well-read *Zeitschrift für die gesamte Strafrechtswissenschaft* also had a soft spot for psychology and picked up on the matter. The two young men also surfed on the back of Hans Gross’s reputation (even though the latter expressed his reservations about the practicality of their method [H. Gross 1905]): when other journals started reporting about Tatbestandsdiagnostik, they almost always highlighted that Wertheimer and Klein were Gross’s students (for example, Stern 1904: 275; Kraus 1905: 58–59; Freud 1906: 3–4).

The visibility of Tatbestandsdiagnostik was helped by a small scandal shortly afterwards: the Swiss psychiatrist Carl Gustav Jung accused them, in a psychiatric journal, of plagiarism. Wertheimer and Klein had been very sparing with references regarding the association method; and, even worse, they seemed to have taken conclusions from Jung’s own work on affective complexes without attribution. It annoyed Jung, who was at this point still building his reputation, that they were now seen as the “inventors” of this method when “a little more piety for their forebearers” would have been appropriate (Jung 1905: 814). Wertheimer protested; Gross soothed; Jung and Wertheimer corresponded. Eventually Jung retracted the accusation, as they agreed that they had independently come to similar findings. Wertheimer and Klein, Jung conceded, had been the first to see the possibility of applying the association method to criminal investigation.^7^

Meanwhile, Tatbestandsdiagnostik had already garnered much attention. The debates sprawled over journals, meetings, and conferences in different fields: law and criminalistics, experimental and applied psychology, psychiatry and psychoanalysis. The conference circuit gave many scholars an opportunity to get acquainted with the method. Experimental psychologists were already warmed up at the first conference for experimental psychology in 1904 in Gießen, where Hans Gross was on the program with a lecture on the subject, although he did not turn up. The matter was discussed at later editions of the same conference, in Frankfurt am Main in 1908 and in Innsbruck in 1910 (Schumann 1904: xiii–xvi; Schumann 1909: 257; Schumann 1911: 104–8). Criminalists and legal scholars could hear about Tatbestandsdiagnostik in Turin at the well-attended sixth international conference for criminal anthropology in 1906 (Brusa 1908: 498) and again, five years later, at its seventh edition in Cologne (Der VII. Internationale 1911); or at the eleventh meeting of the International Union of Penal Law in Brussels in 1910 (Claparède 1910). Even at a more general conference, the 79th meeting of German scientists and medical doctors in Dresden in 1907, the psychiatrist Alfred Hoche spoke about Tatbestandsdiagnostik as an illustration of the increasing resort to scientific methods in psychology (Stadelmann 1908: 462–63).

Those who did not attend conferences could read reports about them, as well as in-depth discussions of and reports of experiments with Tatbestandsdiagnostik, in their preferred journals. The German public prosecutor Alois Zeiler, for instance, reported having read about the method in a journal before trying it out himself a few years later (Zeiler 1944: 79). Gross’s, Aschaffenburg’s, and von Liszt’s journals were the key venues for the debates and included contributions by jurists, psychologists, and psychiatrists. Even beyond these journals, however, there seems not to have been a German-language journal on criminal justice or psychology that did not at least mention the new method.^8^ There were methodological discussions, debates about legal admissibility, comparisons with other techniques, presentations of experiments and case studies. By 1911, when the psychologist Otto Lipmann published a synthesizing monograph on the method, his bibliography contained no less than 80 publications on Tatbestandsdiagnostik—and this did not even include the many short reports and reviews journals published (Lipmann 1911: 89–95).

Even outside the German-speaking world, the new method was noticed, though foreign commentors often struggled to translate the word Tatbestandsdiagnostik.^9^ Legal and psychiatric journals in the Netherlands, for instance, followed the German developments closely, and many Dutch scholars attended German and international conferences.^10^ Interest in the new method was also stimulated through more long-term mobility: in 1904, the German psychiatrist Karl Heilbronner, formerly the senior physician at a psychiatric clinic in Halle, was appointed as a professor in Utrecht. Heilbronner took an interest in Tatbestandsdiagnostik and helped to establish the Dutch “psychiatric-juridical society” in 1907, where many topics that could be classified as forensic psychology were debated. From its incipience, the gatherings of this society several times a year provided occasions for discussions about Tatbestandsdiagnostik.^11^ Inspired by these discussions, two Dutch psychiatrists wrote dissertations about Tatbestandsdiagnostik (Schnitzler 1907; van der Hoeven 1908).

For comparison, the interest was not as profound in the French-speaking world. Like the Dutch, the French were aware that “much has been said in Germany” about this new “legal diagnostic” (Binet 1909: 372). There were brief mentions and some book reviews in legal and general journals, and some critical engagement on the pages of *L’Année psychologique*, the main French journal for experimental psychology edited by Alfred Binet (Claparède 1906: 295–302; Binet 1909). Some legal scholars mentioned the new method and their interventions in international fora show that they were aware of German developments (Granier 1906: 1–4; Varendonck 1914: 118–32), but there were almost no original interventions or experiments in the Francophone scientific world. This is consistent with the overall lesser engagement with experimental psychology in France at the time (Parot 2017: 61). Furthermore, as Binet lamented, the French judiciary was less inclined than its German counterpart to adopt innovative approaches to criminal investigation.^12^

While Tatbestandsdiagnostik gained little traction in Britain, possibly for similar reasons, it sparked great interest in the United States. As in the Netherlands, this popularity was facilitated by the mobility of a German psychologist, in this case Hugo Münsterberg. Münsterberg was a student of Wilhelm Wundt and chaired the psychology laboratory at Harvard University from the 1890s onwards, becoming president of the American Psychological Association in 1898. In 1907, Münsterberg took an interest in forensic psychology. He undertook several experiments, commented on famous cases, and published a series of popular articles. In 1908 followed a book on crime detection and psychology, in which association experiments also figured.^13^ Meanwhile, Münsterberg’s colleagues at the Harvard psychological laboratory published more academic research on the topic (Yerkes & Berry 1909; Kohs 1914; Crane 1915).

Tatbestandsdiagnostik hence quickly made its way through the scientific world. But the goal of Wertheimer and Klein, and of many of their interpellators, was that their knowledge would be used in the practice of criminal justice. For this purpose, it was not enough that psychologists, legal scholars, or even psychiatrists working in the academy engaged with their ideas. They had to reach active magistrates. While some investigating magistrates, lawyers, prosecutors, and practicing psychiatrists attended scientific conferences or read one of the scholarly journals in their field, several scholars undertook attempts to reach those who did not. Some gave lectures for local legal or criminalistic societies. In Munich, for instance, Rudolf Wassermann spoke about Tatbestandsdiagnostik to the local society of jurists in 1907; in Vienna, Adolph Stöhr did the same for the Austrian association of criminalists.^14^ In Berlin, Otto Lipmann taught a special course on psychology for law students in 1908, which gave ample attention to Tatbestandsdiagnostik. A few years later, Karl Marbe taught a similar course in Munich, now specifically for active legal personnel. The texts of these specialized courses were published afterwards, allowing further dissemination (Lipmann 1908: 70–78; Marbe 1913: 61–67). The most popular manuals for investigating judges, with Hans Gross’s manual in the lead (H. Gross 1914: 153–55), discussed Tatbestandsdiagnostik in their pages. Any self-respecting German-speaking judge had the opportunity to acquaint himself with the new technique.

Perhaps more vivid than the lectures and courses were the demonstrations that sometimes accompanied these events, for instance after the lectures in Munich and in Vienna in 1907. In Prague, Hans Gross and Alfred Gross conducted special demonstrations of the method for local professionals, including the chiefs of justice and the police (Kraus 1905: 59; Lederer 1906b: 494–97). Jung held a demonstration for the Legal-Psychiatric Circle in Zurich (Zürcher 1906). During these demonstrations, some secret information was given to a volunteering “suspect.” The fact that the association method lent itself to these public demonstrations may have helped to secure the interest of the judiciary and the police (even though it may have been lackluster in comparison with scientific demonstrations that included intricate psychological instruments). The fact that attendants could witness the success (or failure) of the method themselves increased its validity as an objective and scientific method.

Even beyond psychologists, legal scholars, psychiatrists, magistrates, and police officers, knowledge of Tatbestandsdiagnostik spread to the wider public. Several general newspapers described the method, often in sensational terms, by which people could apparently betray their guilt through a series of simple word reactions.^15^ In the US, the *New York Times* followed the psychological discoveries with interest.^16^ Newspapers also announced public lectures on and demonstrations of this curious new technique to discover criminals. Some lectures were specifically aimed at an interested public: in Düren, for instance, the psychologist Paul Menzerath gave a “popular scientific lecture” about the subject for the local Commercial Association in 1911; while the psychiatrist Cornelis Winkler spoke to the Amsterdam Student Debating Club in 1907.^17^ If Tatbestandsdiagnostik remained a curiosum, the suggestion that it was possible to determine objectively whether someone was guilty of a crime, without them knowing, certainly drew the attention.

This new form of knowledge therefore circulated quite widely. Far from a niche technique, within a decade after its first formulation, Tatbestandsdiagnostik was discussed in several countries and several languages, by people with backgrounds in psychology, psychiatry, law, and criminalistics, and within environments of academics and legal practitioners. Germany, Austria-Hungary, and Switzerland were at the center of the discussions about Tatbestandsdiagnostik. Given the pivotal role German-speaking institutions played in the development of experimental psychology at the turn of the twentieth century, it is unsurprising that attempts to apply this knowledge in practice also originated there (Smith 2013: 77; Wolffram 2018: chap. 2). Tatbestandsdiagnostik circulated so rapidly because journals and conferences that were looking for exactly this type of interdisciplinary and useable knowledge had already been established. Yet Tatbestandsdiagnostik circulated well beyond the German-speaking world, to varying degrees, often in line with the available infrastructure in each region. Heilbronner’s and Münsterberg’s moves to laboratories in the Netherlands and the US, and their instigation of new research and new infrastructure, directly affected the visibility of Tatbestandsdiagnostik in these countries.

## 3. Too Objective and Too Subjective

The quick circulation of knowledge about Tatbestandsdiagnostik was only possible because of the availability of the necessary infrastructure, but the infrastructure alone did not suffice. Tatbestandsdiagnostik was picked up because it responded to several different needs. Wertheimer and Klein formulated their ideas at a moment when experimental psychologists were trying to ensure the continued existence of their field. As Annette Mülberger and Heather Wolffram have shown, experimental psychology was becoming independent from philosophy and some psychologists feared their discipline would not survive on its own. By showing their relevance to society, they might increase their chances of survival and attract new people to their field, as they had with Wertheimer (Wolffram 2020; Mülberger 2009a: 136). Psychologists like Karl Marbe and Hugo Münsterberg hence cited Tatbestandsdiagnostik to show the usefulness of experimental psychology to the world of criminal justice (Marbe 1912: 111; Münsterberg 1908: 7–9).

Since the association method was also popular in psychiatric practice, particularly in psychoanalysis, psychiatrists promoted Tatbestandsdiagnostik as a technique that showed the relevance of *their* discipline, and one that was best performed by them. Jung, as one of the key players in the discussions about Tatbestandsdiagnostik, took a prime role here. But Sigmund Freud himself also argued in a lecture, published afterwards, that the method showed that insights and techniques from psychoanalysis were making their way into criminal justice (Freud 1906: 9).

Tatbestandsdiagnostik not only responded to the needs of experimental psychologists and psychiatrists, but also, and perhaps mainly, to the needs of legal scholars and criminalists. Around 1900, the police was taking over many of the investigative duties examining judges had had in several European countries. Police investigators were less bound by restrictions or duties of impartiality (Lévy 1993). In response, investigating magistrates asserted their superior knowledge, skills, and morals. The lure of “scientific” means of proving crimes was high, surfing on the back of the success of the Sherlock Holmes novels (Burney & Pemberton 2016; Becker 2005). In Germany, specifically, the abolition of the system that predetermined the value of different forms of proof in 1879 had also paved the way for new, scientific means to establish guilt or innocence (Schröer & Niehaus 2006: 216–17; Wolffram 2023: 95–97). Among legal scholars, critique grew of legal dogmatism and traditionalism at the end of the nineteenth century. There was a need for science and empiricism (Mülberger 2009b: 63; Gorphe 1947: 14–15). In 1884, the Italian criminologist Enrico Ferri (1898: 166) argued that criminal trials should use a “scientific, experimental procedure” as much as possible. And this was what Tatbestandsdiagnostik offered. In the words of Hugo Münsterberg, Tatbestandsdiagnostik would usher in an era of criminal investigations that were “swifter and cleaner, more scientific, more humane, and more reliable” (Münsterberg 1908: 109).

The scientific procedure of Tatbestandsdiagnostik held the promise of bypassing a suspect’s will and observing their conscience directly. This was a long-standing problem in criminal justice: since it was against suspects’ interests to confess, a confession could only be expected by somehow circumventing their will. In the past, pain or emotions had been used as methods to accomplish this. But now, a more humane and painless method was presenting itself.^18^ As the Swiss psychologist Édouard Claparède wrote, an association test was almost like an “X-ray to read the brain of another” (Claparède 1910: 516). If the influence of an individual’s will in criminal justice was bypassed, the “ideal criminal investigation” came closer and crime itself would decrease (Lederer 1906b: 488).

For both experimental psychologists and legal scholars, it was therefore important to highlight that the new procedure was “objective.” “By psychological experiments,” the *New York Times* reported in 1907, “we are enabled to bring to the study of mind an almost mathematical certainty” (Discovery Made by a Swiss Doctor 1907). The Dutch psychiatrist Leendert Bouman concurred: “Why are psychological experiments so alluring to the scientifically schooled researcher? In the first place because one hopes to obtain objective results with them” (L. Bouman 1908: 25). Tatbestandsdiagnostik’s popularity was part of the paradigm that privileged “mechanical objectivity” as the standard of proof in science, in justice, and in society overall around 1900: the influence and fallibility of the researcher were reduced by recourse to mechanical instruments with invariable routines (Daston & Galison 2007: 137–39, 256–58; Burney & Pemberton 2016: 18). Early proponents of the method therefore highlighted their adherence to objective and reproducible experimental standards, and disavowed any relation to “metaphysics” or “mysticism” (Jung 1906c: 145–46; Stadelmann 1908: 462–63; Lipmann 1911: 4–5).

And yet, despite the flurry of publications on the subject, there was only one scholar who argued early on that Tatbestandsdiagnostik was ripe for immediate use in practice: Alfred Gross, a student of Hans Gross. In an extensive article in von Liszt’s journal, he detailed why he believed the method was both sufficiently reliable and legally admissible (A. Gross 1906). Most others, even if they were enthusiastic, believed that it was too soon (Stern 1904: 275–76; Kraus 1905: 59–60; see also the comments in H. Gross 1914: 155).

The crux of the many debates that followed was how much objectivity was actually desirable in criminal justice.^19^ Several legal scholars and practitioners were concerned about the possible difference between experimental settings and actual criminal investigations. Early successful experiments were carried out by professors on their students. These students, critics claimed, did not want to fail their professors; and in any case, nothing was at stake if their pretend “crime” was discovered (Lederer 1906b: 493). Moreover, earlier psychological experiments had highlighted the impact of intelligence, gender, and age on associations (A. Gross 1906: 21–22; Hoegel 1907). Yet based on experiments on this very specific sample of the population—mostly young, male, affluent, and by definition educated—psychologists made universalizing claims about the impact of committing a crime on associations.^20^ From their experience, legal scholars and practitioners knew that in real life, unlike the laboratory or the classroom, all sorts of circumstances complicated how people behaved, what knowledge they possessed, and how they felt. People were frightened or perhaps hardened by years of crime, forgot things, could have mental illnesses, or tried to mislead their interrogators. All this affected how and how quickly they would respond to particular words (Stern 1904; Heilbronner 1907: 608, 626; Storfer 1909: 267; Rittershaus 1912: 96–103). Many jurists therefore doubted whether psychological knowledge gained through experiments was at all suitable for criminal justice. All these experiments were useful for science, perhaps, but not for legal practice (Lederer 1906b: 505; Biensfeldt 1908: 458). This view was in line with a general skepsis among practicing magistrates about experimental knowledge. As other historians have shown, they often valued experience in the field much more highly and found that the objectivity of the experiment brought little useful to the courtroom (Wolffram 2018: 224; Mülberger 2009b: 78, 2009a: 144).

Among experimental psychologists and psychiatrists, and some psychologically schooled legal scholars, an opposite critique emerged. Several of these scholars attempted to reproduce or build on the experiments of Wertheimer and Klein. Some of them claimed success: Alfred Gross (sometimes with Hans Gross) in Prague, Franz Kramer and William Stern in Breslau, Éduard Claparède in Geneva, and of course Jung in Zurich (A. Gross 1905: 49; Kramer & Stern 1906; Claparède 1906: 299; Jung 1906b). Other experiments, however, were less successful. Especially in the Netherlands, several failed or inconclusive experiments led experimental psychiatrists to conclude that Tatbestandsdiagnostik was unreliable (Heilbronner 1907; Schnitzler 1907; van der Hoeven 1908; L. Bouman 1908). In turn, this led to reactions, among others from Jung, that the failed experiments were “deplorably superficial” and lacked scientific rigor and experience (Jung 1973a: 518; Rittershaus 1909: 75–82). Wertheimer retorted that “poorly set up experiments prove nothing at all.”^21^ Yet something was clearly amiss. Some scholars wrote that the problem was that Tatbestandsdiagnostik was not objective enough.

These experimental psychologists and psychiatrists especially disapproved of what some perceived as the method’s psychoanalytic elements. Particularly—but not only—Jung was the target of this critique. Karl Heilbronner argued that the main problem lay in how experimenters determined whether or not a reaction word was significant, which often occurred with psychoanalytic theory, if not explicitly then implicitly. Any given word could be seen as a sign of some unconscious mental complex relating to the crime. This made it impossible to reproduce an experiment, and hence the method “lacked any objective basis.”^22^ Leendert Bouman concurred: unlike the earlier statistical association experiments, carried out by Wundt and his students, the method of Tatbestandsdiagnostik lacked objectivity. And this was the method’s worst sin: early psychology might have been subjective, too, “but it seems to me even more questionable when, assuming the appearance of being objective, one allows the subjective to play such an important role” (L. Bouman 1908: 25). The French psychologist Alfred Binet gave the deathblow. “This method of forensic diagnosis is imbued with the same literary and mystical spirit found in the works of Freud and his students,” he wrote. There was no objectivity, no reproducibility, no control, and “a science without control is like a people without morals: it is a beginning of decadence” (Binet 1909: 373).

If any research into Tatbestandsdiagnostik was to follow in the future, experimental psychologists argued, it had to be more strictly on the basis of an objective method (Schnitzler 1907: 98–99). Paul Menzerath, for instance, devised a device to measure reaction times mechanically instead of manually, to ensure greater accuracy (Menzerath 1912: 171). This was also Otto Lipmann’s view when he summarized the debates in 1911. Wary of any mystical psychoanalytical influence, his volume contained extensive series of data, graphs, and formulas pertaining to the different methods of Tatbestandsdiagnostik and the results of his own experiments, in part conducted with Wertheimer. His conclusions, like those of most before him, remained cautious: more experimental research was necessary (Lipmann 1911).

These debates show that in the period up to World War I, almost everyone recognized objectivity, obtained through experimental, reproducible research in a controlled environment, as a key characteristic of Tatbestandsdiagnostik. Indeed, Tatbestandsdiagnostik became a topic around which the virtues and vices of objectivity could be discussed. Whereas many experimental psychologists and psychiatrists believed that its objectivity did not go far enough, legal scholars tended to criticize the lack of practical legal experience in the discussion of the method’s efficacy. If the former wanted more *objectivity* through mechanical experiments, the latter wanted more *subjectivity*, through the personal experience of the investigating officer. The belief in mechanical objectivity was therefore clearly not hegemonic within early-twentieth-century European societies.

## 4. The Decline of Tatbestandsdiagnostik

The extensive experimental studies Lipmann and others had envisaged never materialized. By the beginning of World War I, the heyday of Tatbestandsdiagnostik was coming to an end. At the fifth conference for experimental psychology in 1912, disillusion set in. Karl Marbe still included Tatbestandsdiagnostik in his overview of applications of psychology for other sciences and for practice, but the tone at Paul Menzerath’s lecture on association experiments was more somber. Menzerath argued again that the association method was too dependent on the subjective interpretation of reactions. Moreover, it seemed that hardened criminals were less susceptible to the emotional complexes a crime caused, so the method would not be effective on them. During the discussions, Wertheimer continued to defend the method, but William Stern and the psychiatrist Wilhelm Weygandt agreed with Menzerath (Menzerath 1912). The conference was later remembered as a turning point at which the high expectations for Tatbestandsdiagnostik were lowered (Eliasberg & Jankau 1931: 191). After World War I, few proponents of Tatbestandsdiagnostik remained. Hans Gross, who had inspired and provided a forum for much of this research, died in 1915. Even Max Wertheimer switched to a different topic, becoming famous as one of the founders of Gestalt psychology (Newman 1944).

As a result of its quite sudden rise and fall, the number of actual cases where Tatbestandsdiagnostik was successfully applied in German-speaking areas was negligible. The psychiatrist Otto Mönkemöller, writing in 1930, estimated that the association technique had maybe been applied to one or, at best, a few actual criminal cases (Mönkemöller 1930: 153). Early on, an examining magistrate had asked Alfred Gross to conduct an association test, only to be called off by the prosecutor, who deemed it inappropriate (Lederer 1906a: 163). In 1917, a public prosecutor in Zweibrücken collaborated with an expert to conduct an association test on two suspects in a murder case. The test confirmed the prosecutor’s suspicions, but he declined to use it as evidence in the trial. He feared it would have an adverse effect on a conservative court (Zeiler 1944). I have not found any other instances where the association test was carried out on the initiative of the examining magistrate or prosecutor. This may have several causes: doubts about the reliability, credibility, or legality of the method, aversion to the growing influence of psychological experts in criminal justice, or perhaps wariness of the time-consuming efforts involved in creating, testing, and analyzing the long lists of words.^23^

Still, psychologists and psychiatrists sometimes took the initiative themselves to apply the association method to criminal cases, by making deals with prison or asylum directors or by interpreting their mandate to investigate the sanity of a suspect broadly. In most of these cases, the subjects had already been convicted or had at least confessed.^24^ The psychiatrist Philipp Stein, for instance, studied several criminals, but discussed only one case where the method was useful as proof. In 1909, a woman who denied an abortion betrayed herself during an association test by revealing the name of the doctor who had assisted her. She confessed afterwards (Stein 1909: 196–203). In other cases, an association test was used as part of a range of techniques. Psychiatrist Charles de Montet described a case, also in 1909, in which a “Jungian” word association test had been fruitless, but a free association experiment, in which the suspect had to continue associating words in an endless chain for several hours, led to a confession (due to exhaustion, we might imagine) (Montet 1909: 40–43). Indeed, the main success of the method was that some suspects confessed during or after the associational test, fearing that science had exposed them, even if the test was inconclusive.^25^ Despite this singular advantage, the association method did not catch on in legal practice.

## 5. Afterlife

Although it never regained its former place in the spotlight, some psychologists, psychiatrists, and jurists continued to write about Tatbestandsdiagnostik in the 1920s, 30s, and 40s. Handbooks of forensic psychology, such as Hellwig’s (1927), Mönkemöller’s (1930), or Erismann’s (1947), dutifully but rarely enthusiastically discussed Tatbestandsdiagnostik. Handbooks for criminal investigators, however, did not.^26^ Hans Schneickert even explicitly rejected all psychotechnical methods in his 1926 manual for criminal investigation (Schneickert 1926: 285). Only the posthumous editions of Hans Gross’s manual for criminal investigators, for instance edited by Ernst Seelig in 1941, continued to mention the association method, but even there, the shortcomings and the lack of applications of the technique were highlighted (H. Gross & Seelig 1941: 180–81).

New experimental research was scarce. In the US, there was still some interest in the 1920s, when psychologists Eva Goldstein and Harold Crosland published the results of their laboratory experiments. Crosland claimed success in applying the method to criminal cases (Goldstein 1923; Crosland 1929). However, “the extreme complexity of his statistical calculations,” a colleague later recalled, “was sufficient to frighten away most of the criminologists” (Trovillo 1938: 868). In the German-speaking world, psychologists and jurists sporadically rediscovered Tatbestandsdiagnostik: in 1936, the Swiss psychoanalyst J.B. Lang used the technique to reveal his maid as the thief of his wife’s wallet, while in 1944, the jurist Heinz Gummersbach advertised the possible uses of the technique in criminal justice as if it were a novelty (Lang 1936; Gummersbach 1944).

The times were changing. Increasingly, the objectivity Tatbestandsdiagnostik had promised was no longer desired in criminal justice. In 1927, the psychologically schooled judge Albert Hellwig, echoing earlier colleagues, reasserted the importance of case studies against the dominance of experiments in forensic psychology. Often, he claimed, experience was more useful than experiments (Hellwig 1927: 19–20). Similarly, in his 1935 dissertation, the Dutch forensic psychiatrist Dirk de Bois argued that “in general, one must not value the data obtained by experimental research too highly” (de Bois 1935: 101–3).^27^ His colleague Henri van der Hoeven concurred at a conference three years later: “If an earlier period suffered from a shortage of science, we risk to suffer from an excess” (cited in K. H. Bouman 1938: 67). Scientists were often over-sure of themselves, the legal scholar Bernard Taverne added, bluffing their way to confessions with “scientific” instruments (Taverne 1924: 313, 323–26). The reverence for mathematical certainty had fallen out of fashion. The human mind, several jurists, psychologists, and psychiatrists of the 1920s and 1930s argued, was too complicated for simple experimental studies.

Insofar as forensic psychologists were still praising Tatbestandsdiagnostik, they primarily highlighted its subjective side rather than its objective characteristics. Had the psychoanalytic elements of Tatbestandsdiagnostik been the subject of critique around 1910, most of its remaining supporters were convinced that these links were precisely what made it a useful tool. Psychiatrists Wladimir Eliasberg and V. Jankau, for instance, argued in 1931 that Tatbestandsdiagnostik was particularly apt to study a suspect’s consciousness of their crime, including their feelings and mental state, in line with psychoanalytic methods, rather than the objective fact of committing the crime (Eliasberg & Jankau 1931). Perhaps the association method could not provide definite objective proof, the French legal scholar François Gorphe concluded in 1947, but it was a useful “scientific procedure for discovering or clarifying psychological clues, which then still need to be interpreted” (Gorphe 1947: 96). Numbers and measurements, it seemed, meant little without feelings and interpretations.

Those who continued to believe in mechanical objectivity in criminal justice found their way to different methods. They abandoned word associations in favor of measuring the suspect’s physical emotional responses to what an interlocutor or they themselves said, including their breath, heartrate, blood pressure, and the electric activity of their skin. As we have seen, this approach was not new: already in the late nineteenth century, Lombroso and others had experimented with applying psychodiagnostic instruments on criminals. Wertheimer and Klein discussed these instruments as well, and some psychologists had proposed to combine them with the word association test (Lang 1936). Enthusiasm for using these instruments was growing in the 1920s, in Europe, but particularly elsewhere (e.g., Seelig 1927; see also Bachhiesl 2013). In the Soviet Union, for instance, Alexander Luria combined measuring reaction times with measuring hand tremors, claiming that this approach led to “greater precision and objectivity” in the experiments (Luria 1930: 154). More famously and more radically, by the end of the 1920s, some of the techniques associated with Tatbestandsdiagnostik developed into the “lie detector” in the USA, applicable in criminal justice and just about anywhere else (Alder 2007; Bunn 2012).

In Europe, meanwhile, one staunch supporter of the association method remained: Carl Gustav Jung. In his early work on Tatbestandsdiagnostik, Jung had declared an allegiance to pure experimental psychology in line with mechanical objectivity (Jung 1906c). However, because of the key place Jung accorded to unconscious complexes, Jung’s approach to the technique required more interpretation than strict experimental psychologists would allow. Jung’s approach to Tatbestandsdiagnostik was psychoanalytic and interpretative; he referred more to his personal experience with criminals than to objective measurements. This becomes clear from a crucial part of Jung’s argumentation. While Hans Näf’s reactions were slow and aberrant in response to critical terms, it could be that this was due to fear, since Näf already knew exactly what he was accused of (a key problem critics of Tatbestandsdiagnostik’s application in legal practice had also observed). However, Jung claimed that “experience” shows that innocent people are more affected by general accusations, whereas guilty people focus more on specific details. Näf was in the latter situation (Jung 1973b: 538–39). The experiment only gained meaning because of Jung’s experience. The objective facts were interpreted with subjective means.

**Fig. 2:**
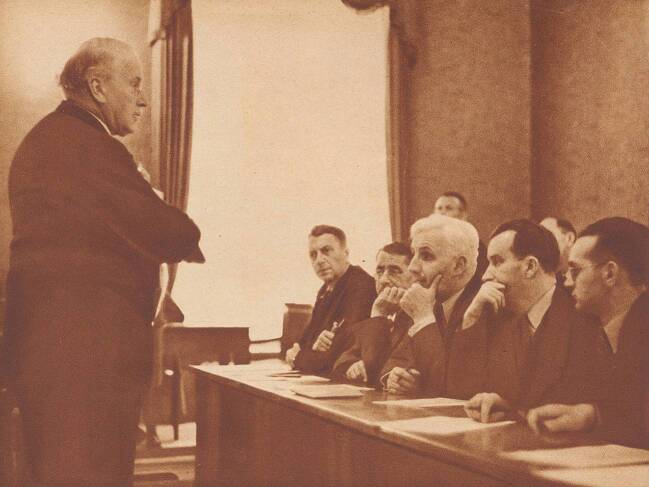
Jung addresses the jury in the Näf case during the 1938 retrial. From *Zürcher Illustrierte*, no. 49, December 2, 1938, p. 1498

So, while Näf was initially convicted, it should not surprise us that a retrial followed a few years later. This time, Jung’s pride would suffer. He appeared again as an expert witness (Fig. 2), but the defense now also brought in another expert: the young psychologist and phenomenologist Hans Kunz. Kunz argued that, despite Jung’s “experience,” it was possible that Näf’s reactions were the result of fear and of the extensive preliminary investigations and psychiatric evaluations. Jung continued to defend his thesis, but had to qualify his conclusions. Consequently, whereas his expertise “had made a great impression to the detriment of the defendant” in the first trial, it had much less of an impact during the retrial (Baechi 1940: 9). Näf was found innocent. While the case did not only rest on Jung’s experiments, it shows that jurors were at least not entirely convinced of the reliability of the method. Jung’s approach to Tatbestandsdiagnostik comes closest to what Daston and Galison have called “trained judgment”: machine registrations were interpreted by the experienced scientist. But in the case of Tatbestandsdiagnostik, this clearly did not catch on. The method that had once heralded the march towards objectivity lost its flavor when the limits of that objectivity were too starkly visible.

## Conclusions

The Näf trial is, as far as I have been able to find, the only time the results of an association test were presented as evidence of guilt in a European criminal court. In the mid-1930s, it came more than three decades after psychologists and lawyers had started thinking about Tatbestandsdiagnostik, and two decades after interest in the method had started to wane. This late and not quite effective application of the method is indicative of its contentiousness. As a case study in the history of knowledge, Tatbestandsdiagnostik’s brief career shows the importance of infrastructure in the initial circulation of knowledge. Around 1900, a burgeoning infrastructure, including journals, courses, laboratories, and conferences, was in place to accommodate just the type of knowledge that Tatbestandsdiagnostik offered. This infrastructure, as well as the individual mobility this infrastructure encouraged, facilitated a quick circulation not only within the German-speaking world, but also beyond. But infrastructure did not suffice to make Tatbestandsdiagnostik into a success in the longer term.

The history of Tatbestandsdiagnostik reveals a series of tensions that plagued early-twentieth-century European knowledge cultures: tensions between knowledge gained through experiments and knowledge based on experience; tensions between belief in the superiority of mechanical objectivity and the subjective appreciation of evidence that dominated in European criminal courts; tensions between theoretical scientific knowledge and everyday life. Tatbestandsdiagnostik was so enticing in its early years because it provided the opportunity to discuss these tensions, while also holding the promise of resolving a number of them. Tatbestandsdiagnostik would supplement experience with experiment and bring objective knowledge to the subjective truth-finding practices of criminal justice. To do so, it would apply scientific insights in real-life situations. This was attractive to many of the people involved: for experimental psychologists, this was a means to show the utility of their methods and findings for solving real-life problems; for jurists, a way to highlight the modernity and reliability of their investigative practices.

Ultimately, however, as positions became more entrenched, the tensions could not be resolved. Many experimental psychologists had difficulties with the subjective elements of Tatbestandsdiagnostik, while, after the initial curiosity in the first years of the twentieth century, many jurists became disenchanted by the crudeness of objective psychological insights. Especially after World War I, jurists reasserted the value of knowledge gained through extensive practice. Psychological experiments, they argued, were too far removed from the reality of criminal investigation. While criminal justice could accept strict mechanical objectivity (such as chemical analysis) and intuitive human judgments, relying on the trained judgment of psychologists proved to be more difficult.

The impact of Tatbestandsdiagnostik on the practice of criminal investigation hence remained limited. But that does not mean there was no impact at all. The high visibility of Tatbestandsdiagnostik for a little under a decade led to important new collaborations. Already in 1912, the psychiatrist Ernst Rittershaus, who was skeptical about the applicability of Tatbestandsdiagnostik in practice, observed that the debates had contributed to extensive exchanges between jurists, physicians, psychologists, and psychiatrists. “Perhaps the greatest and most important success of the method,” Rittershaus (1912: 104) concluded, “lies in the promotion of a kind of mutual influence.”

One way this influence manifested itself was through the proliferation of the idea that people could betray themselves without even knowing it. Through the discussions about Tatbestandsdiagnostik, psychologists, psychiatrists, jurists, and the wider public were increasingly acquainted with the idea that scientific techniques could reveal “unconscious” psychic matter (Mezger 1919: 156–60). They promoted a sense of a fragmented self, stable and deep, but not wholly in control of itself.^28^ In later applications of similar techniques, such as the polygraph, this idea would shift: the polygraph was not expected to reveal unconscious complexes, but the subject’s intention to deceive (Bunn 2012). What remained, however, was the idea that science had a role to play in revealing the psyche without the suspect’s will.

## Footnotes

1 For my reconstruction of the case, I rely on the reports of Spiegel (1937) and Baechi (1940). See also the reconstruction in Jung & Neumann (2015: 81–82n254).

2 E.g., Adam (2020). See for similar nuances Ruberg (2023a, b).

3 For calls to do this, see Secord (2004); Östling et al. (2018).

4 Wilhelm Weygandt estimated that there were about eight such societies in 1912 (Marbe 1912: 112). One example is the Psychologisch-forensische Vereinigung, established in Göttingen in 1903.

5 All we know is that, after studying law in Prague, he managed Wertheimer’s legal affairs in Czechoslovakia after the latter had moved to America in 1933 (Michael Wertheimer et al. 1992: n. 27).

6 Respectively the *Archiv für Kriminal-Anthropologie und Kriminalistik*; the *Beiträge zur Psychologie der Aussage*, becoming the *Zeitschrift für angewandte Psychologie* in 1907; and the *Monatsschrift für Kriminalpsychologie und Strafrechtsreform*.

7 The altercation is extensively described in Michael Wertheimer et al. (1992).

8 The issue was covered in, among others and apart from the journals mentioned above, *Archiv für die gesamte Psychologie, Archiv für Strafrecht und Strafprozeß, Centralblatt für Nervenheilkunde und Psychiatrie, Der Gerichtssaal, Juristisch-Psychiatrische Grenzfragen, *and *Schweizerische Zeitschrift für Strafrecht*.

9 Literally, Tatbestandsdiagnostik would translate as something along the lines of “the diagnostics of (criminal) facts,” but observers everywhere outside Germany considered the word to be untranslatable. It is “one of those words without meaning, at least for us, that only the Germans can coin,” an Italian editor commented (Jung 1973a: 615n2).

10 Starting in 1905, the main journal on criminal justice regularly reported on Tatbestandsdiagnostik in its “Notices from foreign journals”: Mededeelingen (1905); Mededeelingen (1906); Mededeelingen (1907).

11 E.g., Leendert Bouman’s lecture on the subject in 1907, later published as L. Bouman (1908).

12 Binet cited in Nicolas, Gounden, and Sanitioso 2014: n. 4. The lesser interest among French jurists was probably partly due to differences in the legal systems.

13 Münsterberg (1908: chap. 3). On Münsterberg and his role in establishing forensic psychology in the US, see Hale (1980); Spillmann and Spillmann (1993: 329–30).

14 *Allgemeine Zeitung (München)*, nr. 518, November 8, 1907, p. 5 (via Digipress); Stransky (1907).

15 E.g., Grabowsky (1905); Isserlin (1906). See also the announcements of lectures and demonstrations cited above.

16 Discovery Made by a Swiss Doctor (1907). See also the articles and satirical comments discussed in Bunn (2012: chap. 3).

17 *Dürener Zeitung*, nr. 59 (March 17, 1911) (via Zeitpunkt.nrw); *Het Nieuws van den Dag* (April 24, 1907): 2; *Algemeen Handelsblad* (April 14, 1907): 9; *Leeuwarder Courant *(April 24, 1907): 6; *Bredasche Courant* (April 24, 1907) (via Delpher).

18 Münsterberg (1908: 73–76); Werner (1910: 527); Mezger (1919: 155). See also Hofman (2021: 38).

19 Apart from this, there were also extensive debates on whether the method would be legally admissible: see a.o. Hoegel (1907: 31); Lipmann (1911: 83–85); Balla (1936).

20 The students were presumably also mostly white, but this characteristic was not discussed in this context.

21 Wertheimer’s response in Menzerath (1912: 174). See also Groß (1907: 212).

22 Heilbronner (1907: 605–6). Similar views in Schnitzler (1907: 60).

23 On the tensions between magistrates and psychological experts, see Wolffram (2018). On the extensive efforts needed to prepare the test, see Discovery Made by a Swiss Doctor (1907). On the practical legal difficulties, see Mezger (1919: 156).

24 Stein (1909); Heilbronner (1907); Schnitzler (1907). See also some instances described in Herbold-Wootten (1982: 249–50).

25 This was the result of the experiment described in Erismann (1947: 118–20). Criminalists were aware of this: H. Gross & Seelig (1941: 181).

26 Tatbestandsdiagnostik is absent from e.g., Anuschat (1921); Lichem (1935); Meinert (1939).

27 De Blois added that “Experimental psychological research into depth and size [of the human psyche] with our current tools is much like measuring the contents of the sea with a measuring glass.”

28 On these different dimensions of selfhood, see Hofman (2016).

## References

[CR1] Adam, Alison 2020. Crime and the Construction of Forensic Objectivity from 1850: Introduction. In: Alison Adam (ed.). *Crime and the Construction of Forensic Objectivity from 1850*. Cham: Springer: 1–13**.**

[CR2] Alder, Ken 2007. *The Lie Detectors: The History of an American Obsession*. New York: The Free Press.

[CR3] Anuschat, Erich 1921. *Die Gedankenarbeit des Kriminalisten Kriminalist. Schlußfolgerungskunst u. ihre Grenzen*. Berlin: S. Gerstmann.

[CR4] Bachhiesl, Christian 2013. The Search for Truth by “Registration of Expression” – Polygraph Experiments in Graz in the 1920s. *European Polygraph* (7:2): 55–68.

[CR5] Baechi, W. 1940. Der Mordfall Näf – Zürich. Verurteilung im Schwurgerichtsverfahren. Freispruch und 25 000 Franken Entschädigung im Wiederaufnahmeverfahren. *Archiv für Kriminologie (Kriminal-Anthropologie und Kriminalistik)* (107): 5–13, 93–97, 119–20.

[CR6] Balla, Karl 1936. *Tatbestandsdiagnostische Methoden und ihre strafprozessuale Zulässigkeit*. Emsdetten: H. & J. Lechte.

[CR7] Becker, Peter 2005. *Dem Täter auf der Spur: eine Geschichte der Kriminalistik*. Darmstadt: Primus.

[CR8] Biensfeldt, J. 1908. Kriminalistische Vereinigung Erlangen. Über psychologische Tatbestandsdiagnostik. Vortrag von Dr. Specht. *Monatsschrift für Kriminalpsychologie und Strafrechtsreform* (4): 457–58.

[CR9] Binet, Alfred 1909. Le diagnostic judiciaire par la méthode des associations. *L’Année psychologique* (16:1): 372–83.

[CR10] Bois, Dirk Wilhelm de. 1935. *Bijdrage tot de kennis der forensische psychiatrie*. PhD Thesis, Rijksuniversiteit Leiden.

[CR11] Bouman, K. H. (ed.) 1938. *Verslag van den criminologendag gehouden op Zaterdag 26 November 1938 in het Wilhelmina Gasthuis te Amsterdam*. Amsterdam: Van Rossen.

[CR12] Bouman, Leendert 1908. Experimenteel-psychologisch onderzoek naar een begane handeling. *Psychiatrische en neurologische bladen* (12): 24–44.

[CR13] Brusa, Emilio 1908. De la valeur psychologique du témoignage. In: *Comptes rendus du VIe Congrès international d’anthropologie criminelle (Turin, 28 avril–3 mai 1906)*, 494–500. Turin: Bocca.

[CR14] Bunn, Geoffrey C. 2012. *The Truth Machine: A Social History of the Lie Detector*. Baltimore: Johns Hopkins University Press.

[CR15] Bunn, Geoffrey C. 2019. “Supposing That Truth Is a Woman, What Then?”: The Lie Detector, the Love Machine, and the Logic of Fantasy. *History of the Human Sciences* (32:5): 135–63.

[CR16] Burney, Ian and Neil Pemberton 2016. *Murder and the Making of English CSI*. Baltimore: Johns Hopkins University Press.

[CR17] Claparède, Édouard 1906. La psychologie judiciaire. *L’année psychologique* (12): 295–302.

[CR18] Claparède, Édouard 1910. Psychologie du témoignage. *Bulletin de l’Union internationale de droit pénal* (17): 496–518.

[CR19] Crane, Harry W. 1915. A Study in Association Reaction and Reaction Time. With an Attempted Application of Results in Determining the Presence of Guilty Knowledge. *The Psychological Monographs* (18:4): 1–75.

[CR20] Crosland, H.R. 1929. *The Psychological Methods of Word-Association and Reaction-Time as Tests of Deception*. University of Oregon.

[CR21] Danziger, Kurt 1980. Wundt’s Psychological Experiment in the Light of His Philosophy of Science. *Psychological Research* (42:1): 109–22.

[CR22] Daston, Lorraine and Peter Galison 2007. *Objectivity*. New York: Zone Books.

[CR23] Der VII. Internationale Kongreß für Kriminal-Anthropologie. (Vierter Verhandlungstag). *Kölnische Zeitung* (nr. 1122, October 13, 1911).

[CR24] Discovery Made by a Swiss Doctor May Play an Important Part in Criminal Trials. *The New York Times* (June 9, 1907): sec. P.

[CR25] Dror, Otniel Yizhak 1999. The Scientific Image of Emotion: Experience and Technologies of Inscription. *Configurations* (7:3): 355–401.

[CR26] Eliasberg, W. and V. Jankau 1931. Zur forensischen Bedeutung des Assoziationsexperimentes. *Zeitschrift für die gesamte Strafrechtswissenschaft* (51): 191–98.

[CR27] Erismann, Theodor 1947. *Psychologie und Recht*. Bern: A. Francke.

[CR28] Ferri, Enrico 1898. *Criminal Sociology*. New York: D. Appleton.

[CR29] Freud, Sigmund 1906. Tatbestandsdiagnostik und Psychoanalyse. *Archiv für Kriminal-Anthropologie und Kriminalistik* (26): 1–10.

[CR30] Goldstein, Eva R. 1923. Reaction Times and the Consciousness of Deception. *The American Journal of Psychology* (34:4): 562–81.

[CR31] Gorphe, François 1947. *L’appréciation des preuves en justice: essai d’une méthode technique*. Paris: Sirey.

[CR32] Grabowsky, Adolph. Psychologische Tatbestandsdiagnostik. *Allgemeine Zeitung (München)* (no. 289, December 15, 1905): 497–99 (accessed via Digipress).

[CR33] Granier, Camille 1906. *Aveu et témoignage. Critique de la preuve orale. Extrait du Journal du Ministère public*. Paris: Marchal et Billard.

[CR34] Greve, Ylva 2004. *Verbrechen und Krankheit: die Entdeckung der Criminalpsychologie im 19. Jahrhundert*. Cologne: Böhlau.

[CR35] Gross, Alfred 1905. Zur psychologischen Tatbestandsdiagnostik. *Monatsschrift für Kriminalpsychologie und Strafrechtsreform* (2): 182–84.

[CR36] Gross, Alfred 1906. Die Assoziationsmethode im Strafprozeß. *Zeitschrift für die gesamte Strafrechtswissenschaft* (26): 19–40.

[CR37] Gross, Alfred 1907. Die Assoziationsmethode im Strafprozeß. Kriminalpsychologische Studie. *Zeitschrift für die gesamte Strafrechtswissenschaft* (27): 175–212.

[CR38] Gross, Hans 1905. Zur psychologischen Tatbestandsdiagnostik. *Archiv für Kriminal-Anthropologie und Kriminalistik* (19): 49–59.

[CR39] Gross, Hans 1914. *Handbuch für Untersuchungsrichter als System der Kriminalistik*. *Teil 1*. 6th ed., 2 vols. Munich: J. Schweitzer.

[CR40] Gross, Hans and Ernst Seelig 1941. *Handbuch der Kriminalistik*. 8th ed. Berlin: J. Schweizer.

[CR41] Gummersbach, Heinz 1944. Psychologische Geständnisse. Ein Beitrag zur Methodik der Diagnose des Tatbestandes. *Kriminalistik: Monatshefte für die gesamte kriminalistische Wissenschaft und Praxis* (18): 26–28.

[CR42] Hale, Matthew Jr. 1980. *Human Science and Social Order: Hugo Münsterberg and the Origins of Applied Psychology*. Philadelphia: Temple University Press.

[CR43] Heilbronner, Karl 1907. Die Grundlagen der “psychologischen Tatbestandsdiagnostik”. Nebst einem praktischen Fall. *Zeitschrift für die gesamte Strafrechtswissenschaft* (27): 601–56.

[CR44] Hellwig, Albert 1927. *Psychologie und Vernehmungstechnik bei Tatbestands-Ermittlungen: eine Einführung in die forensische Psychologie für Polizeibeamte, Richter, Staatsanwälte, Sachverständige und Laienrichter*. Berlin: Langenscheidt.

[CR45] Herbold-Wootten, H. 1982. The German Tatbestandsdiagnostik: A Historical Review of the Beginnings of Scientific Lie Detection in Germany. *Polygraph* (11:3): 246–57.

[CR46] Hoegel 1907. Die “Tatbestandsdiagnostik” im Strafverfahren. *Monatsschrift für Kriminalpsychologie und Strafrechtsreform* (4): 26–31.

[CR47] Hoeven, Henri van der 1908. *De invloed der affectieve meerwaarde van voorstellingen in het woordredaktie-experiment*. ’s-Hertogenbosch: Henri Berger.

[CR48] Hofman, Elwin 2016. How to Do the History of the Self. *History of the Human Sciences* (29:3): 8–24.

[CR49] Hofman, Elwin 2021. *Trials of the Self: Murder, Mayhem and the Remaking of the Mind, 1750–1830*. Manchester: Manchester University Press.

[CR50] Hofman, Elwin 2022. A Useful Science: Criminal Interrogation and the Turn to Psychology in Germany around 1800. *Journal of the History of the Behavioral Sciences* (58:3): 319–34. 35253239 10.1002/jhbs.22193

[CR51] Horn, David G. 2003. *The Criminal Body: Lombroso and the Anatomy of Deviance*. London: Routledge.

[CR52] Isserlin, Max. Psychologie und Verbrecherüberführung. *Kölnische Zeitung* (Erste Beilage zur Sonntags-Ausgabe, nr. 291, March 18, 1906, accessed via Zeitpunkt.nrw).

[CR53] Jung, Carl Gustav 1905. Zur psychologischen Tatbestandsdiagnostik. *Centralblatt für Nervenheilkunde und Psychiatrie* (28): 813–15.

[CR54] Jung, Carl Gustav 1906a. *Diagnostische Assoziationsstudien: Beiträge zur experimentelle Psychopathologie. Vol. 1*. 2 vols. Leipzig: J.A. Barth.

[CR55] Jung, Carl Gustav 1906b. Die psychologische Diagnose des Tatbestandes. *Juristisch-psychiatirsche Grenzfragen. Zwanglose Abhandlungen* (4:2): 3–47.

[CR56] Jung, Carl Gustav 1906c. Die psychopathologische Bedeutung des Assoziationsexperimentes. *Archiv für Kriminal-Anthropologie und Kriminalistik* (22): 145–62.

[CR57] Jung, Carl Gustav 1937. Zur psychologischen Tatbestandsdiagnostik: Das Tatbestandsexperiment im Schwurgerichtsprozess Näf. *Archiv für Kriminologie (Kriminal-Anthropologie und Kriminalistik)* (100): 123–30.

[CR58] Jung, Carl Gustav 1973a. New Aspects of Criminal Psychology. In: *Collected Works of C.G. Jung, Volume 2: Experimental Researches.* Princeton: Princeton University Press: 518–25.

[CR59] Jung, Carl Gustav 1973b. On the Psychological Diagnosis of Evidence: The Evidence-Experiment in the Näf Trial. In: *Collected Works of C.G. Jung, Volume 2: Experimental Researches*. Princeton: Princeton University Press: 532–39.

[CR60] Jung, Carl Gustav and Erich Neumann. 2015. *Analytical Psychology in Exile: The Correspondence of C.G. Jung and Erich Neumann*. Martin Liebscher (ed.). Princeton: Princeton University Press.

[CR61] Kohs, Samuel C. 1914. The Association Method in Its Relation to the Complex and Complex Indicators. *The American Journal of Psychology* (25:4): 544–94.

[CR62] Kramer, Franz and William Stern 1906. Selbstverrat durch Assoziation. Experimentelle Untersuchungen. *Beiträge zur Psychologie der Aussage* (2:4): 457–88.

[CR63] Kraus, O. 1905. Psychologische Tatbestandsdiagnostik. *Monatsschrift für Kriminalpsychologie und Strafrechtsreform* (2): 58–61.

[CR64] Lang, J.B. 1936. Experimentelle Beiträge zur Psychologischen Diagnose des Tatbestandes. *Zentralblatt für Psychotherapie und ihre Grenzgebiete einschliesslich der medizinischen Psychologie und psychischen Hygiene* (9:2): 104–21, 151–77.

[CR65] Lederer, Max 1906a. Die Verwendung der psychologischen Tatbestandsdiagnostik in der Strafrechtspraxis. *Monatsschrift für Kriminalpsychologie und Strafrechtsreform* (3): 163–72.

[CR66] Lederer, Max 1906b. Zur Frage der psychologischen Tatbestandsdiagnostik. *Zeitschrift für die gesamte Strafrechtswissenschaft* (26): 488–506.

[CR67] Lévy, René 1993. Police and the Judiciary in France since the Nineteenth Century: The Decline of the Examining Magistrate. *British Journal of Criminology* (33:2): 167–86.

[CR68] Lichem, Arnold 1935. *Die Kriminalpolizei: Handbuch für den kriminellen Polizeidienst*. 2nd ed. Graz.

[CR69] Lipmann, Otto 1908. *Grundriss der Psychologie für Juristen*. Leipzig: J.A. Barth.

[CR70] Lipmann, Otto 1911. *Die Spuren interessebetonter Erlebnisse und ihre Symptome. Theorie, Methoden und Ergebnisse der “Tatbestandsdiagnostik”.* Leipzig: J.A. Barth.

[CR71] Lück, Christian and Michael Niehaus 2007. Pathologie des Geständnisses. Zum Stellenwert von Selbstaussagen um 1900. In: Jo Reichertz and Manfred Schneider (eds.). *Sozialgeschichte des Geständnisses: zum Wandel der Geständniskultur*. Wiesbaden: VS Verlag für Sozialwissenschaften: 143–70.

[CR72] Luria, A.R. 1930. Die Methode der abbildenden Motorik in der Tatbestanddiagnostik. *Zeitschrift für angewandte Psychologie* (35): 139–83.

[CR73] Marbe, Karl 1912. Die Bedeutung der Psychologie für die übrigen Wissenschaften und die Praxis. In: F. Schumann (ed.). *Bericht über den V. Kongreß für experimentelle Psychologie in Berlin vom 16. bis 20. April 1912*. Leipzig: J.A. Barth: 110–16.

[CR74] Marbe, Karl 1913. *Grundzüge der forensischen psychologie*. Munich: Beck.

[CR75] Mededeelingen uit buitenlandsche tijdschriften. 1905. *Tijdschrift voor Strafrecht* 17: 429–88.

[CR76] Mededeelingen uit buitenlandsche tijdschriften. 1906. *Tijdschrift voor Strafrecht* 18: 264–312.

[CR77] Mededeelingen uit buitenlandsche tijdschriften. 1907. *Tijdschrift voor Strafrecht* 19: 245–88.

[CR78] Meinert, Franz 1939. *Vernehmungstechnik*. Lübeck: Verlag für polizeiliches Fachschrifttum.

[CR79] Menzerath, Paul 1912. Die sogenannten Komplexmerkmale beim Assoziationsexperiment. In: F. Schumann (ed.). *Bericht über den V. Kongreß für experimentelle Psychologie in Berlin vom 16. bis 20. April 1912*. Leipzig: J.A. Barth: 170–75.

[CR80] Mezger, Edmund 1919. Die Beschuldigtenvernehmung auf psychologischer Grundlage. *Zeitschrift für die gesamte Strafrechtswissenschaft* (40): 152–68.

[CR81] Mönkemöller, Otto 1930. *Psychologie und Psychopathologie der Aussage*. Heidelberg: Winter.

[CR82] Montet, Ch. de 1909. Assoziationsexperimente an einem kriminellen Fall. *Monatsschrift für Kriminalpsychologie und Strafrechtsreform* (6): 37–47.

[CR83] Mülberger, Annette 2009a. Karl Marbe und die Anwendung der Psychologie im Rechtswesen vor dem ersten Weltkrieg. In: Mathias Schmoeckel (ed.). *Psychologie als Argument in der juristischen Literatur des Kaiserreichs*. Baden-Baden: Nomos: 133–52.

[CR84] Mülberger, Annette 2009b. Teaching Psychology to Jurists: Initiatives and Reactions Prior to World War I. *History of Psychology* (12:2): 60–86. 19831235 10.1037/a0015993

[CR85] Mülberger, Annette 2017. Mental Association: Testing Individual Differences before Binet. *Journal of the History of the Behavioral Sciences* (53:2): 176–98. 28236298 10.1002/jhbs.21850 PMC5413820

[CR86] Münsterberg, Hugo 1908. *On the Witness Stand: Essays on Psychology and Crime*. New York: Doubleday.

[CR87] Newman, Edwin B. 1944. Max Wertheimer: 1880–1943. *The American Journal of Psychology* (57:3): 428–35.

[CR89] Nicolas, Serge, Juan Segui, and Ludovic Ferrand 2000a. L’Année Psychologique: History of the Founding of a 100-Year-Old French Journal. *History of Psychology* (3:1): 44–61. 11624163 10.1037/1093-4510.3.1.44

[CR90] Nicolas, Serge, Juan Segui, and Ludovic Ferrand 2000b. Les premières revues de psychologie: la place de L’Année Psychologique. *L’Année psychologique* (100:1): 71–110.

[CR88] Nicolas, Serge, Yannick Gounden, and Rasyid Bo Sanitioso 2014. Alfred Binet, Founder of the Science of Testimony and Psycho-Legal Science. *L’année Psychologique* (114:2): 209–29.

[CR91] Östling, Johan, Erling Sandmo, David Larsson Heidenblad, Anna Nilsson Hammar and Kari Nordberg (eds.) 2018. *Circulation of Knowledge: Explorations in the History of Knowledge*. Lund: Nordic Academic Press.

[CR92] Parot, Françoise 2017. *La psychologie française dans l’impasse: du positivisme de Piéron au personnalisme de Fraisse*. Paris: Editions matériologiques. 10.3138/cbmh.34.2.567 28920746

[CR93] Rittershaus, Ernst 1909. Die Komplexforschung (“Tatbestandsdiagnostik”). *Journal für Psychologie und Neurologie* (15): 61–83, 184–220, (16): 1–43.

[CR94] Rittershaus, Ernst 1912. Psychologische Tatbestandsdiagnostik. (Die sogenannte “Strafuntersuchung der Zukunft”). *Jahrbücher der hamburgischen Staatskrankenanstalten* (17): 85–104.

[CR95] Ruberg, Willemijn 2023a. Hysteria as a Shape-Shifting Forensic Psychiatric Diagnosis in the Netherlands c. 1885–1960. *Gender & History* (35:2): 565–81.

[CR96] Ruberg, Willemijn 2023b. Introduction. In: Willemijn Ruberg, Lara Bergers, Pauline Dirven, and Sara Serrano Martínez (eds.). *Forensic Cultures in Modern Europe*. Manchester: Manchester University Press: 1–24.

[CR97] Schmoeckel, Mathias 2009. Der Einfluss der Psychologie auf die Entwicklung des Zeugenbeweises im 19. und beginnenden 20. Jahrhundert. In: Mathias Schmoeckel (ed.). *Psychologie als Argument in der juristischen Literatur des Kaiserreichs*. Baden-Baden: Nomos: 57–85.

[CR98] Schneickert, Hans 1926. *Kriminaltaktik und Kriminaltechnik*. Die Kriminalpolizei, Bd. 2. Hamburg: Lübeck.

[CR99] Schnitzler, Johann Gustav 1907. *Onderzoekingen over de diagnostiek van voorstellings-complexen met behulp van het associatie-experiment*. Utrecht: Wentzel.

[CR100] Schröer, Norbert and Michael Niehaus 2006. Geständnismotivierung als edukative Beziehungsarbeit. *Kriminologisches Journal* (38:3): 210–27.

[CR101] Schumann, F. (ed.) 1904. Bericht über den I. Kongreß für experimentelle Psychologie. Leipzig: J.A. Barth.

[CR102] Schumann, F. (ed.) 1909. Bericht über den III. Kongress für experimentelle Psychologie. Leipzig: J.A. Barth.

[CR103] Schumann, F. (ed.), 1911 Bericht über den IV. Kongress für experimentelle Psychologie. Leipzig: J.A. Barth.

[CR104] Secord, James A. 2004. Knowledge in Transit. *Isis* (95:4): 654–72. 16011300 10.1086/430657

[CR105] Seelig, Ernst 1927. Die Registrierung unwillkürlicher Ausdrucksbewegungen als forensisch-psychodiagnostische Methode. *Zeitschrift für angewandte Psychologie* 28: 45–84.

[CR106] Smith, Roger 2013. *Between Mind and Nature: A History of Psychology*. London: Reaktion Books.

[CR107] Spiegel, Hans Wilhelm 1937. Der Fall Näf. Mord und Versicherungsbetrug, Selbstmord oder Unfall? *Archiv für Kriminologie (Kriminal-Anthropologie und Kriminalistik)* (100): 98–122.

[CR108] Spillmann, Jutta and Lothar Spillmann 1993. The Rise and Fall of Hugo Münsterberg. *Journal of the History of the Behavioral Sciences* (29:4): 322–38.

[CR109] Stadelmann, Heinrich 1908. Die Psychologie auf der 79. Versammlung deutscher Naturforscher und Ärzte zu Dresden. *Zeitschrift für angewandte Psychologie und psychologische Sammelforschung* (1): 462–65.

[CR110] Stein, Philipp 1909. Tatbestandsdiagnostische Versuche bei Untersuchungsgefangenen. *Zeitschrift für Psychologie* (52): 161–237.

[CR111] Stern, William 1904. Psychologische Tatbestandsdiagnostik. *Beiträge zur Psychologie der Aussage* (2:2): 275–77.

[CR112] Storfer, Adolf 1909. Ein Gefahr der kriminalpsychologischen Tatbestandsdiagnostik. *Schweizerische Zeitschrift für Strafrecht* (22): 266–76.

[CR113] Stransky, Erwin 1907. Österreichsche kriminalistische Vereinigung. Sitzungen in Wien. *Monatsschrift für Kriminalpsychologie und Strafrechtsreform* (4): 316–22.

[CR114] Taverne, B.M. 1924. Iets over de plaats van aanwijzingen en rapporten van deskundigen in ons wettelijk bewijssysteem. *Tijdschrift voor Strafrecht* (34): 307–26.

[CR115] Trovillo, Paul V. 1938. A History of Lie Detection. *Journal of the American Institute of Criminal Law and Criminology* (29): 848–81, (30): 104–19.

[CR116] Varendonck, Julien 1914. *La psychologie du témoignage*. Ghent: Maison d’éditions & d’impression.

[CR117] Werner, Georges 1910. Psychologie du témoignage. *Bulletin de l’Union internationale de droit pénal* (17): 518–32.

[CR118] Wertheimer, Max and Julius Klein 1904. Psychologische Tatbestandsdiagnostik. Ideen zu psychologisch-experimentellen Methoden zum Zwecke der Feststellung der Anteilnahme eines Menschen an einem Tatbestande. *Archiv für Kriminal-Anthropologie und Kriminalistik* (15): 72–113.

[CR119] Wertheimer, Michael, D. Brett King, Mark A. Peckler, Scott Raney, and Roddy W. Schaef 1992. Carl Jung and Max Wertheimer on a Priority Issue. *Journal of the History of the Behavioral Sciences* (28:1): 45–56. 11612657 10.1002/1520-6696(199201)28:1<45::aid-jhbs2300280104>3.0.co;2-p

[CR120] Wolffram, Heather 2018. *Forensic Psychology in Germany: Witnessing Crime, 1880–1939*. Basingstoke: Palgrave Macmillan.

[CR121] Wolffram, Heather 2020. Forensic Psychology in Historical Perspective. In: Wade E. Pickren (ed.). *Oxford Research Encyclopedia of Psychology*. Oxford: Oxford University Press.

[CR122] Wolffram, Heather 2023. Teaching Grossian Criminalistics in Imperial Germany. In: Willemijn Ruberg, Lara Bergers, Pauline Dirven, and Sara Serrano Martínez (eds.). *Forensic Cultures in Modern Europe*. Manchester: Manchester University Press: 92–116.

[CR123] Word Clues to Crimes: Dr. Jung on His System. *The Daily Mail* (October 9, 1935).

[CR124] Yerkes, Robert M. and Charles S. Berry 1909. The Association Reaction Method of Mental Diagnosis (Tatbestandsdiagnostik). *The American Journal of Psychology* (20:1): 22–37.

[CR125] Zeiler, Alois 1944. Ein Versuch mit der “Assoziationsmethode”. *Archiv für die gesamte Psychologie* (112): 77–82.

[CR126] Zürcher, Max 1906. Zur psychologischen Diagnose des Tatbestandes. *Monatsschrift für Kriminalpsychologie und Strafrechtsreform* (3): 163–72.

